# Watch Out! Magnetoencephalographic Evidence for Early Modulation of Attention Orienting by Fearful Gaze Cueing

**DOI:** 10.1371/journal.pone.0050499

**Published:** 2012-11-29

**Authors:** Fanny Lachat, Teresa Farroni, Nathalie George

**Affiliations:** 1 Université Pierre et Marie Curie-Paris 6, Centre de Recherche de l’Institut du Cerveau et de la Moelle épinière, UMR-S975, and Centre MEG-EEG, Paris, France; 2 CNRS, UMR 7225, CRICM, Paris, France; 3 Inserm, U 975, CRICM, Paris, France; 4 Centre for Brain and Cognitive Development, Birkbeck College, University of London, London, United Kingdom; 5 Dipartimento di Psicologia dello Sviluppo e della Socializzazione, University of Padua, Padua, Italy; Macquarie University, Australia

## Abstract

Others’ gaze and emotional facial expression are important cues for the process of attention orienting. Here, we investigated with magnetoencephalography (MEG) whether the combination of averted gaze and fearful expression may elicit a selectively early effect of attention orienting on the brain responses to targets. We used the direction of gaze of centrally presented fearful and happy faces as the spatial attention orienting cue in a Posner-like paradigm where the subjects had to detect a target checkerboard presented at gazed-at (valid trials) or non gazed-at (invalid trials) locations of the screen. We showed that the combination of averted gaze and fearful expression resulted in a very early attention orienting effect in the form of additional parietal activity between 55 and 70 ms for the valid versus invalid targets following fearful gaze cues. No such effect was obtained for the targets following happy gaze cues. This early cue-target validity effect selective of fearful gaze cues involved the left superior parietal region and the left lateral middle occipital region. These findings provide the first evidence for an effect of attention orienting induced by fearful gaze in the time range of C1. In doing so, they demonstrate the selective impact of combined gaze and fearful expression cues in the process of attention orienting.

## Introduction

Humans are fundamentally social animals. Adaptive behaviour thus involves the decoding and integration of the many social – verbal as well as non verbal – signals sent by others. The human face is an essential source of such signals. In particular, eye gaze and emotional expression are essential cues in non verbal communication [1]–[3]. In everyday interpersonal interactions, the integrated processing of these cues allows deciphering the mental states and intentions of others. For instance, seeing someone suddenly gazing at a nearby point in space with a fearful expression may be interpreted as signalling a potential threat at this location, whereas seeing someone gazing alike but with a smiling face may be construed as a signal for a nearby likeable object. Thus the meaning of a given facial signal, here gaze, is dependent on other accompanying information, such as emotional expression. In the case of attentional processes elicited by gaze, it is likely that the influence of gaze on attention varies with the associated emotion. How does the integration of gaze and emotion cues impact on the processes of attention orienting induced by gaze? Although there have been numerous studies on attention processes related to gaze perception [4–13 see 14], extremely little is known about the integration of emotion and gaze in attention orienting.

Indeed, overall, few studies investigated how the human brain integrates emotion and gaze and very few of them focused on attention orienting processes. The studies that investigated the integration of emotion and gaze cues from faces examined the influence of gaze direction on the processing of emotional expression. For example, Adams and Kleck [15], [16] and Sander et al. [17] demonstrated that gaze direction modulates the perception of emotional expression, with direct gaze facilitating the processing and enhancing the perception of approach-related emotions (e.g., happiness and anger) and averted gaze facilitating the processing of avoidance-related emotions (e.g. fear and sadness). This behavioral pattern of interaction was associated with activations in the amygdala and superior temporal sulcus, which are known to be involved in the perception of both facial expression and gaze (e.g. [18]–[20]; see also [21]). In addition, one study examined the influence of facial emotional expression on the perception of the direction of gaze; it demonstrated that angry faces tend to be perceived as direct gaze faces over wider range of gaze deviation angles than fearful and neutral faces do ([22]). The neural underpinnings of the integration of gaze and emotion cues in the process of attention orienting, however, remain largely unexplored.

Gaze is a powerful cue to orient an observer’s attention. Seeing someone gazing at a peripheral location in the environment results in the alignment of the perceiver’s attention with that of the gazer (so-called ‘joint attention’). This phenomenon has been traditionally investigated with Posner-like paradigms in which a leftward or rightward deviated gaze serves as the central cue for attention orienting. This gaze cue is followed by a target displayed either at the gazed-at location (valid targets) or at the opposite side (invalid targets). The typical cue-to-target time interval (SOA) in those paradigms is comprised between 200 and 700 ms [14]. Valid targets are detected, discriminated and identified more rapidly than invalid targets [10], [23], [24]. As illustrated in the example above, it is likely that the attentional effect of gaze may be influenced by the emotional expression of the gazing face. In particular, fearful expression, as a typical reaction to threatening events, may be expected to enhance the orienting of attention, hence the cueing effect, induced by gaze. However, at the behavioral level, somewhat mitigated results have been obtained: only a few studies demonstrated an enhancement of the gaze cueing effect by associated fearful expression in classical paradigms of attention cueing by gaze [25], [26], whereas the others did not report any such significant increase [27]–[29] or found such enhancement in high-anxious subjects only [30]–[32]; the influence of fearful expression on gaze cueing effect seemed more reliably observed from derived version of the classical paradigm [33], [34]. These contrasted results may be related to the difficulty of capturing such behavioral effect in laboratory setting. Here, we wanted to investigate the interaction between gaze and emotion on attention orienting at the neural level, in order to test whether there may be a selective, early attention orienting effect induced by fearful gaze.

There have been many studies on the influence of attention on stimulus processing [for recent reviews see 35–37]. For long, these studies seemed to indicate that P1– which typically peaks between 90 and 120 ms in response to visual stimuli – was the first evoked potential to reflect the impact of attention on the neural processing of target stimuli [38]–[42]. Such attentional modulation of the P1 was also highlighted in attention cueing paradigms using neutral face gaze as the attention orienting cue [8], [9], [43], [44]. However, an increasing number of studies have revealed earlier modulation of brain responses by attention over the recent years [45]–[48]. These studies showed that attention influences brain responses in the time range of the C1, which peaks between 50 and 90 ms and involves sources in the striate and extrastriate cortices. For example, Poghosyan and Ioannides [47] and Kelly et al. [45] showed that the C1 is affected by attention as early as from 55 ms after the stimulus onset. These findings suggest that relevant cues may trigger extremely early influence of attention on brain responses to targets. In this respect, considering the significance of the coupling between an averted gaze and a fearful expression, it may be expected that the combination of fearful expression with gaze direction elicits a selectively early attention effect as compared to gaze cues accompanied by neutral or happy expression. The aim of our study was to test this hypothesis: We investigated the interaction between emotional expression and the orienting of attention induced by gaze in order to examine if an early effect of attention orienting may be elicited by fearful gaze.

We designed a Posner-like attention orienting paradigm where the gaze of fearful and happy faces served as the central cue to orient attention toward checkerboard targets, in a simple target detection task. This protocol was used in a study with magnetoencephalography (MEG), which enables the recording of brain activity at a millisecond time scale. We focused our analysis on the first 100 ms of target processing in order to examine the attentional effect triggered by fearful – as compared to happy – gaze cues in the time range of the C1 in response to the target stimuli. We found an early (55–70 ms) effect of attention orienting induced by fearful gaze, which involved sources in the regions of the left superior parietal lobule and lateral middle occipital cortex.

## Materials and Methods

### Ethics Statement

All the participants gave written informed consent to participate in the study. The protocol was approved by the local ethics committee (CPP Ile-de-France VI, protocol nb. 07024). The participants received money compensation for their participation.

### Participants

Fifteen right-handed, healthy volunteers (6 female; age range = 19–34 years, mean age = 27.2±1.1 years) participated in the study. All participants had normal or corrected-to-normal vision and were naïve to the purpose of the experience. After the experiment, they completed the Spielberg State-Trait of Anxiety Inventory (Spielberg, 1983). The mean of their score for the state anxiety was 26.3 (standard error of the mean, SEM = 3.8). Thus, according to the manual of the French version of the STAI, the participants were categorized as low anxious (mean of the score in the corresponding population: 34.8).

### Stimuli

Sixteen faces (9 female) with direct gaze, each under happy, fearful and neutral expressions, were selected from the Karolinska Directed Emotional Faces series (Lundqvist et al., 1998). Photoshop 7.0 (Adobe systems, San Jose, CA) was used to centre the pictures in the middle of an oval frame, so that non facial contours were excluded and the eyes fell on the horizontal midline of the frame. Then, the images were manipulated to change the gaze direction of the original pictures. Gaze was averted to the left for half of the pictures and to the right for the other half. The eye sclera of every face, including the original pictures under direct gaze, was colored homogeneously in light grey. Mirror symmetry was used to create the missing side of gaze aversion, for a total of 144 different pictures (16 different face identities, each under 3 facial expressions: neutral, fearful, happy, and 3 gaze directions: direct, averted to the right, averted to the left).

We checked that there was no difference in luminance between fearful (2.5±0.3 Cd/m^2^) and happy faces (2.6±0.3 Cd/m^2^) (Student t-test: t(15) = −0.58, p = 0.13). All faces subtended a visual angle of 4° horizontally and 7° vertically (corresponding to a size of 236 x 320 pixels).

The target stimulus was a black and white checkerboard comprising 6 rows and 3 columns. It subtended a visual angle of 0.4° by 0.2°.

All stimuli were presented on a grey background.

### Design and Procedure

Participant were seated in a dimly lit, sound-proof, magnetically shielded room, with a screen placed at a viewing distance of 85 cm. Stimuli were delivered by a PC computer using a home-made software. They were backprojected onto the screen through a video projector (Mitsubishi x120) housed outside the magnetically shielded room and two mirrors inside the room.

**Figure 1:**
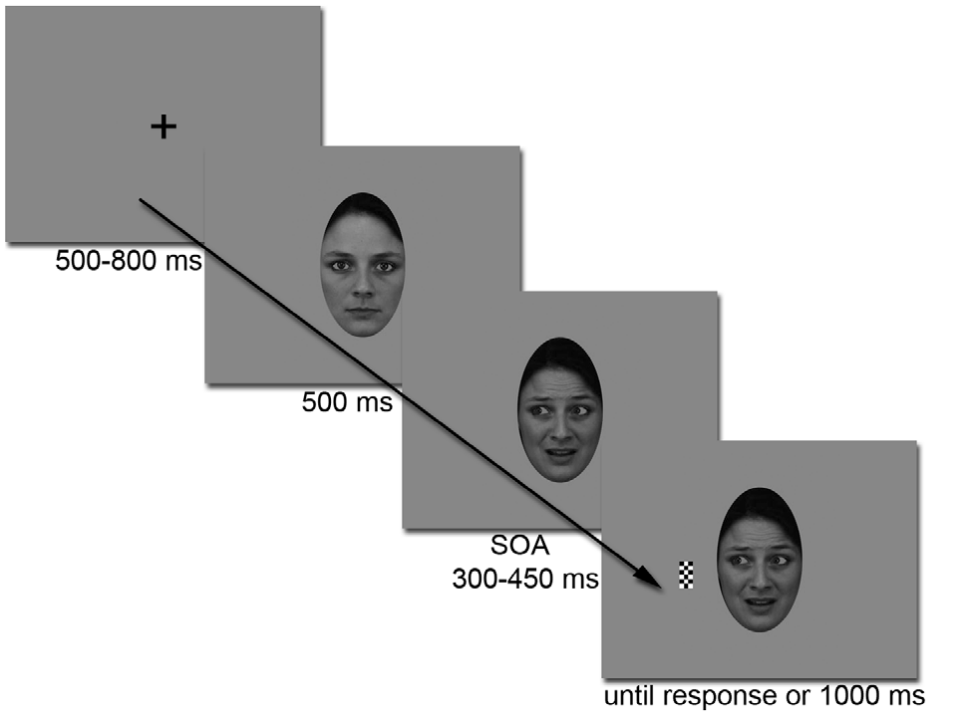
Example of a fear congruent trial. Each trial started with the presentation of a fixation cross in the center of the screen for 500 to 800 ms. A neutral face gazing at the participant was then presented for 500 ms. Then, the emotion of the face changed to either a fearful (here) or a happy expression, and the eyes were simultaneously averted either to the right or to the left side of the screen, or remained straight ahead. After a variable SOA of 300 to 450 ms, the target checkerboard appeared either on the right or on the left side of the screen. The subject’s task was to respond to the target as fast as possible. The target and the face stayed on screen until the subject’s response or during 1 s maximum.

Each trial began with the presentation of a central fixation cross for 500 to 800 ms. The participant was required to fixate on this cross and not to move his/her eyes throughout the blocks of trials. The fixation cross was replaced by a neutral face with direct gaze, which appeared in the centre of the screen so that the fixation point was located midway between the eyes. After 500 ms, the neutral face was replaced by the same individual’s portrait but with a happy or fearful expression and gazing to the left or right side of the screen or still straight ahead. Then, following a variable time interval (stimulus onset asynchrony, SOA) of 300, 350, 400 or 450 ms, the checkerboard target appeared on the right or left of the face, at a visual angle of 5.8° from the centre of the screen, while the face still remained on display. We used a varying delay between the gaze cue and the target onset in order to reduce cerebral activities that can be associated with target anticipation, and to cancel out any remaining magnetic activities time-locked to the preceding emotional gaze cue beyond about 300 ms; this allows yielding a stable baseline in the event-related fields time-locked to the target onset (see [49] for a similar approach). The participants were required to press a button response as soon as they detected the target appearance while keeping their eyes firmly fixed between the eyes of the faces. Participants were aware of the fact that the gaze did not predict the location of the incoming target. Reaction times were recorded. To discourage anticipatory responses, catch trials were included where the checkerboard target was absent and the subject was instructed not to respond. The target and the face remained present until the response of the participant or 1000 ms had elapsed. The next trial started after a 1000 ms inter-trial delay, allowing time for the participants to blink between trials (Figure 1).

There was a total of 768 trials corresponding to 4 repetitions of each of the 16 different individual faces under each of the 12 conditions of emotions (fear/happy) by gaze directions (left/right/direct) by target sides (right/left), plus 96 catch trials (16 per emotion and gaze direction). These trials were randomly distributed in 8 blocks, so that every individual face was seen once under each condition of emotion and gaze-target validity (valid when the gaze pointed to the target location/invalid when the gaze pointed to the side opposite to the target location/uncued when the gaze remained direct whatever the target location) in each block. An additional eye movement calibration block was included so as to allow computing the correspondence between EOG signal (in µV) and eye movements (in degrees of visual angle).

### MEG Acquisition

The experiment was conducted at the MEG centre of the Centre de Neuroimagerie de Recherche (CENIR), Paris, France. Magnetic fields were recorded with a 151 DC-Squid whole-head system (Omega 151, CTF Systems, Port Coquitlam, B.C., Canada) at a sampling rate of 1250 Hz (band-pass filter 0 to 200 Hz). Seventeen external reference gradiometers and magnetometers were included to apply a synthetic third-gradient to all MEG signals for ambient field correction. Three external coils were attached to reference landmarks on the participant (left and right preauricular points, plus nasion) in order to control his/her head position at the beginning of each block and reposition it if needed. Eye movements (electrooculograms, EOG) were recorded by bipolar electrodes, with two electrodes placed at the outher canthi of the eyes for the horizontal eye movements, and two electrodes placed above and below the right eye for the vertical eye movements. The recording also included the signal of a photodiode that detected the actual appearance of the stimuli on the screen within the MEG room. This allowed correcting for the delay introduced by the video projector (20 ms) and averaging event-related magnetic fields (ERFs) precisely time-locked on the actual onset of the target stimulus.

### ERF Analysis

Trials with saccades (rejection threshold: 1° of visual angle from fixation), eye blinks or muscle artifact were rejected upon visual inspection of the MEG and eyetracking signals. The data were time locked to −100 ms before and 200 ms after the appearance of the target, baseline corrected according to the 100 ms preceding the target onset, and digitally low-pass filtered at 40 Hz. Averages were computed separately for each condition of emotion (happy/fearful), gaze-target validity (valid/invalid/uncued), and target side (right/left). In addition, since a marker was inserted at the time where the target should have appeared (at random SOA of 300 to 450 ms) in the catch trials, averages were also computed in response to the ‘absent target’ following happy and fearful faces in these trials. Finally, grand averages of ERFs were computed across subjects.

### Measurements and Statistical Analyses

Trials with RT shorter than 150 ms and longer than 1000 ms were excluded from the analyses as well as non responded target trials (mean omission rate = 3.8±4.9%; range = 0–19%). When we analysed ERFs to catch trials, those trials where the subjects made a button press were excluded from the analysis as well (mean false alarm rate = 2.6±0.6%; range = 0–8%).

In classical gaze cueing paradigms, there is no uncued condition since such condition would be biased by the fact that it lacks a temporal landmark in comparison with cued conditions. This bias did not apply here as there was an additional cue (the facial expression change) simultaneous to the gaze cue. Thus our design included an uncued condition, where the facial expression changed to happy or fearful but the gaze remained direct. In order to check that this condition did not account for our findings, we analysed the factor of gaze-target validity in two ways: with the 3 (valid, invalid and uncued) conditions included as well as with only the valid and invalid conditions entered in the analysis. This did not change any of the results observed. For the sake of simplicity, we chose to present the most classical analysis contrasting only valid with invalid conditions (see table 1 for the statistical analysis with the 3-levels factor of gaze-target validity).

**Table 1:** Grand mean reaction time (and standard error of the mean) for each emotion and for each cue-target validity conditions.

	Valid (ms)	Invalid (ms)	Gaze cueing effect (ms)
**Fearful**	315 (12)	328 (12)	13
**Happy**	316 (12)	328 (11)	12
**Mean**	**315(12)**	**328 (12)**	**13**

The resulting gaze cueing effect (RT difference between invalid and valid trials) is indicated in the rightmost column.

For the behavioural data analysis, the participant’s RT and accuracy were measured and submitted to repeated-measures analyses of variance (ANOVA) with Gaze-target validity (Valid/Invalid), Emotion (Happy/Fear) and Target side (Right/Left) as within-subject factors.

For the ERF analysis, we measured the mean amplitude of the ERFs in the early time window (55–70 ms) of the M70, the magnetic equivalent of the C1 over 5 left parietal sensors (MLC15, MLT14, MLP13, MLP33, MLP34). This measure was submitted to a repeated-measures ANOVA with emotion, gaze-target validity and target side (right/left) as within-subject factors.

All statistical analyses were performed with Statistica 7 (Statsoft, Inc.) software.

### Source Localisation

Data analysis was performed with Brainstorm [50], which is documented and freely available for download online under the GNU general public license (http://neuroimage.usc.edu/brainstorm). Cortical current source density mapping was obtained using a distributed source model consisting in 15,000 current dipoles in each subject and condition. Dipole locations and orientations were constrained to the cortical mantle of a generic brain model derived from the standard MNI template brain of the Montreal Neurological Institute, provided in the BrainStorm’s distribution. This head model was warped to the standard geometry of the MEG sensor cap. MEG forward modelling was computed with the overlapping-spheres analytical model. Cortical current maps were then computed from the MEG time series in response to the target under each condition of emotion (happy/fearful) and gaze-target validity (valid/invalid), using a linear inverse estimator, the weighted minimum norm current estimate (WMNE).

We focused our analysis on the 55–70 ms time window. We computed the difference of the cortical currents for valid versus invalid trials, under each emotion condition, and we ran paired Student t-tests contrasting the valid and invalid conditions for fearful gaze cues, at each time point and every vertex of the cortical current source density maps. These differences and contrasts were performed between −50 and +100 ms for display purpose, but our analysis focused on the 55–70 ms time window. We also averaged the difference maps of the cortical currents between 55 and 70 ms to obtain the mean source activity related to the effect of attention in this time window. We combined three threshold criteria to determine significantly activated regions: Only sources showing a mean activity difference between valid and invalid conditions of at least 3 pA.m (about 40% of the maximum activity) between 55 and 70 ms and for which the uncorrected p threshold was inferior to.001 with an extent threshold over at least 10 vertices in the 55–70 ms time window were considered as significant. We further checked that there was no significant early source activity obtained in the contrast between valid and invalid trials for the happy gaze cues.

## Results

### Behavioural Results

The classical gaze cueing effect was found with faster response times (RT) for validly cued targets (mean = 315±12 ms) as compared to invalidly cued targets (mean = 328±12 ms) (F(1,14) = 25.1, p<0.0002). This effect did not depend on the side of the target (F<1). There was no interaction between gaze-target validity and emotion (F<1), and the mean RT difference between valid and invalid trials was similar for fearful and happy faces (STable 1). There was not any effect of emotion (F<1).

### Evoked Magnetic Fields

**Figure 2:**
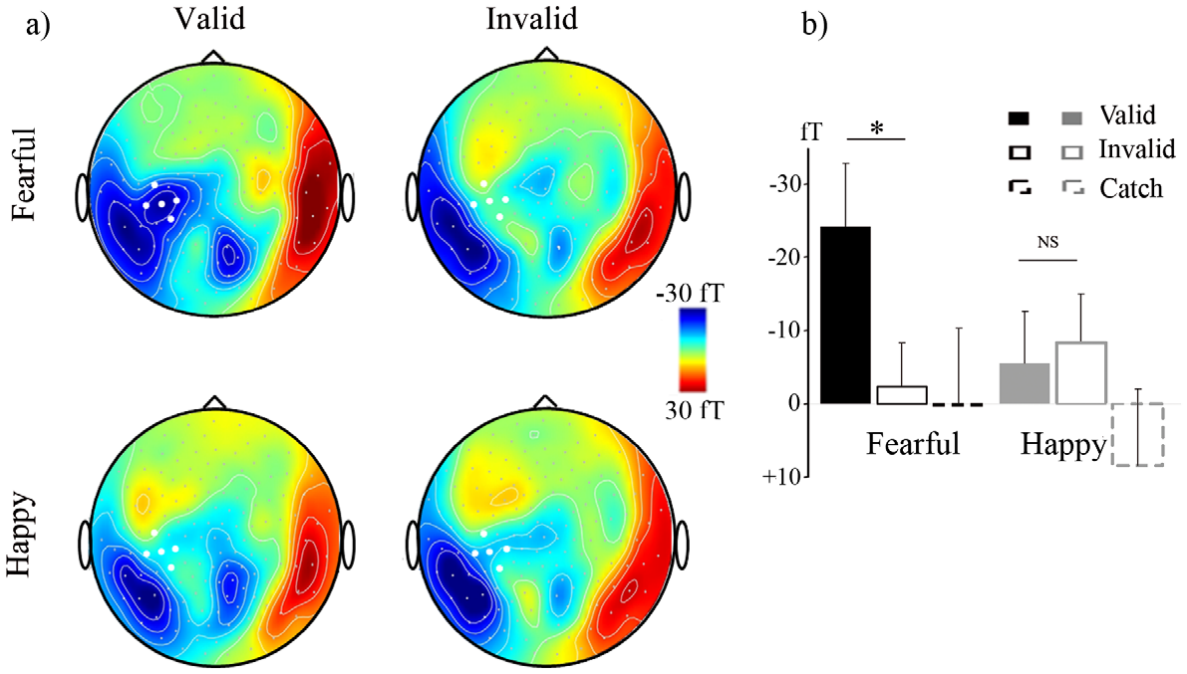
Early (55–70 ms) attention orienting effect induced fearful gaze. a) Grand mean maps of the mean amplitude of ERF in response to targets between 55 and 70 ms, under the four experimental conditions of cue-target validity and emotion. The white dots represent the 5 parietal sensors of measurement. b) Bar plots of the mean amplitude of ERFs on the five sensors of measurement between 55 and 70 ms, for valid, invalid, and catch trials following fearful and happy gaze cues. The bars represent grand mean values, and the lines represent the standard error of the mean.

Our analysis focused on the first 100 milliseconds of target processing. The magnetic equivalent of the C1 (so-called M70) rose from about 50 ms over posterior sensors and reached a maximum between 70 and 85 ms depending on sensors. In the early time window of the M70, data examination suggested that there was an additional activity on the left posterior parietal regions selective of the targets validly cued by a fearful gaze (Figure 2). Thus, we focused our analysis on this activity, as observed on the grand mean of the ERFs: we measured the mean amplitude of magnetic responses between 55 and 70 ms on five parietal sensors centred on this activity.

**Figure 3:**
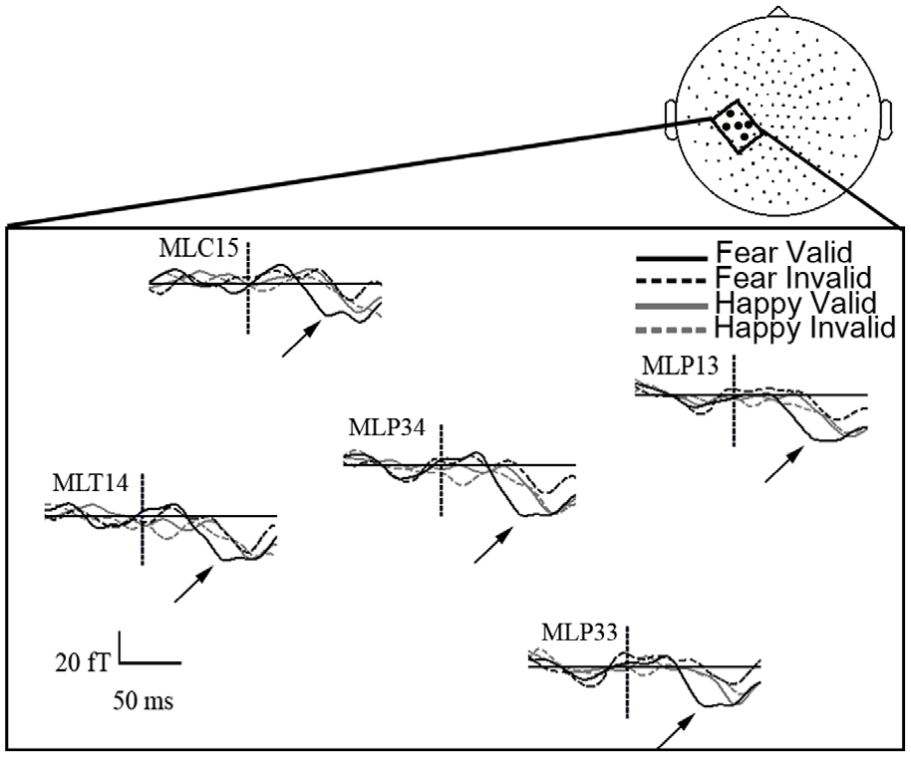
Time course of the attention orienting effect induced fearful gaze. The time course of the grand-averaged ERFs is represented on the five sensors of interest, under the 4 experimental conditions of cue-target validity and emotion.

Statistical analysis confirmed that valid relative to invalid targets elicited a greater left posterior parietal activity between 55 and 70 ms post-target onset (F(1,14) = 6.70, P = 0.02). This effect did not depend on the side of the target (F<1; see also Figure 1). However, it was qualified by an interaction with the emotion of the face (F(1,14) = 5.44, p<.05). Planned comparisons revealed that this early validity effect was selective of fearful face cues (F(1,14) = 11.72, p<0.005; for happy face cues: F<1). As can be seen on Figure 3, it seemed that fearful gaze cues induced a selective attention orienting effect in the form of earlier magnetic responses to the valid target over parietal sensors in the early time range of the M70.

In addition, in order to check if there was any other attention effect in our time window of interest, we performed an ANOVA on the mean amplitude of magnetic responses between 55 and 70 ms considering every sensor, with Gaze-target validity (Valid/Invalid), Emotion (Happy/Fear) and Target side (Right/Left) as within-subject factors. This confirmed the effect observed on parietal sensors and revealed an additional main effect of validity on a right occipital sensor set (see Figure 1). The interaction between validity and emotion was not significant in this latter region. Follow-up planned comparisons of the effect of validity in the fearful and happy gaze cueing conditions respectively, indicated that the effect of validity was significant in the fearful gaze cueing condition on the left parietal sensor region as well as on one right occipital sensor (MRO22), whereas it did not reach significance on any sensor in the happy gaze cueing condition. Thus, this analysis confirmed the left parietal effect reported above while indicating no other reliable effect of attention in our time window of analysis.

Could this effect be related to the residual activity from the cue rather than to the target processing? This appears unlikely as it was an effect of cue-target validity; this factor was instantiated only at the target appearance. However, to test more formally this possibility, we split the data according to the SOA (that is the delay between the emotional face gaze cue and the target onset). If the effect of cue-target validity was time-locked to the emotional face gaze cue, then it should vary with the SOA. We thus ran an ANOVA with SOA as an additional within-subject factor. Although this analysis was based on data of lower signal-to-noise ratio due to the limited number of trials per condition, it confirmed that the effect of cue-target validity was not dependent on the SOA insofar as the interactions between validity and SOA, between emotion and SOA as well as between validity, emotion and SOA were not significant (all F<1, all p>0.7). Moreover, we examined magnetic responses to catch trials. If the early effect observed was elicited by the fearful gaze cues as such, then it should be visible in the catch trials following those cues (relative to the happy gaze cues). Yet, there was no hint of an early additional parietal activity in the catch trials (Figure 2 and Figure 2). Finally, there was not any hint of a parietal activity in response to targets following the fearful direct gaze cues either (see Figure 2). Altogether, these results confirmed that the early parietal activity observed reflected an influence of attention on target processing that was selective of fearful (relative to happy) gaze cues.

### Source Localisation

**Figure 4:**
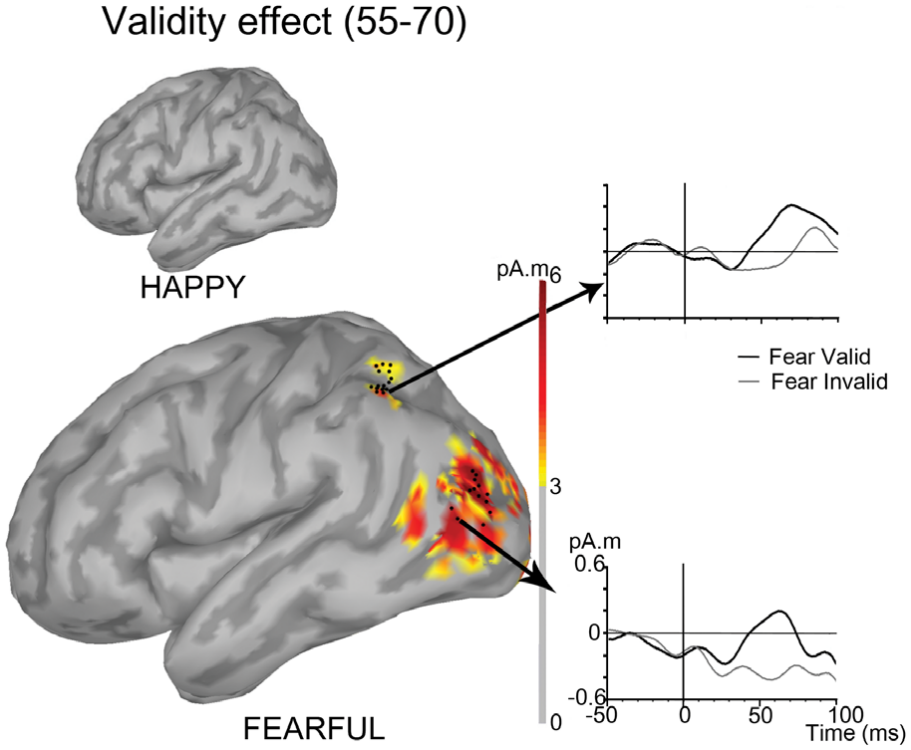
Source localization of the early attention orienting effect induced by fearful gaze cues. The grand average of the mean source amplitude difference between the valid and the invalid conditions in the 55–70 ms time window are projected on a left lateral view of a template brain. Only sources with a mean amplitude of source activation difference above 3 pA.m are represented. The black dots represent the cortical sources where the cue-target validity effect reached significance (p<.001; 15 vertices in the left superior parietal lobule region and 13 vertices in the left lateral middle occipital gyrus region). The smaller brain on the top illustrates the absence of significant activation in response to valid versus invalid targets in the same time window for happy gaze cues. On the right: Grand mean time course of the vertex showing the maximal source amplitude difference for valid versus invalid targets following fearful gaze cues.

We then turned to source localization in order to examine the brain regions that were selectively activated in response to valid relative to invalid targets following fearful gaze cues in the early time window identified. Thus we estimated the cortical sources activated by valid and invalid targets following fearful gaze cues, and we computed the mean source amplitude difference between these two conditions in the 55–70 ms time window. Statistical analysis of the cortical current source density maps showed that two brain regions were significantly activated in the contrast of valid versus invalid trials for fearful gaze cues between 55 and 70 ms (t(14)>4.15, p<.001 over at least 10 vertices in the 55–70 ms time window, in both regions): The first region comprised 15 significant vertices and was centred in the region of the left superior parietal lobule; the second region comprised 13 significant vertices and was centred in the region of the left lateral middle occipital gyrus (Figure 4 and Table 2). There was not any source activation that reached significance for valid versus invalid trials in the happy gaze cues condition.

**Table 2:** Talairach coordinates (in mm) of the vertex showing the maximum difference of source activity for valid versus invalid targets following fearful gaze cues, in the parietal and occipital regions.

REGION	x	y	Z
**Superior parietal lobule**	−36	−61	51
**Lateral middle occipital**	−38	−87	10

## Discussion

This study tested the hypothesis that the combination of a fearful expression with an averted gaze cue may result in very early attention orienting effect as assessed by magnetic brain responses to cued versus uncued targets. In agreement with this hypothesis, we found an influence of attention between 55 and 70 ms following the target onset, which was selective of fearful (relative to happy) face cues. Source localisation showed that this effect involved left posterior parietal and lateral occipital regions.

Our finding brings the first experimental evidence for a very early influence of attention induced by fearful gaze. In doing so, it adds to the growing number of recent studies which have shown that attention may influence the brain responses to targets in the time range of the C1 [45]–[48]. Our study extends these findings by showing that a complex social cue, namely fearful gaze, can also induce an attention orienting effect in the time range of C1. Previous studies using neutral face gaze cues reported influences of the cueing of attention by gaze in later time ranges only, affecting the P1 or even later component [8], [9], [11], [43], [44]. Thus, our finding supports the view that the combination of gaze with a threat-related facial expression elicits a selectively early attention influence on target processing. This result fits with the significance of fearful gaze as signalling a potential threat in the environment. It seems that the signal of attention conveyed by the eyes direction and the emotional signal conveyed by the facial expression are integrated early in the subsequent processing of targets. Following a recent study in patients with unilateral temporal lobectomy [51] it may be speculated that this integration involves the amygdala which may then feed back to posterior visual regions and parietal regions to trigger amplification gain mechanisms for the processing of valid versus invalid targets.

Only one group has previously investigated the neural correlates of the interaction between gaze cueing and emotion on attention orienting, with mitigated results. Fichtenholtz et al [52] used dynamic pictures of an Ekman and Friesen’s emotional face. The face displayed happy or fearful expression and gazed at or away an emotionally salient, positive or negative, target. The subject’s task was to identify the emotionally laden content of the target. In a follow-up study [53], the same authors used fearful and neutral faces which gazed at or away checkerboards that could be oriented either vertically or horizontally. The subject’s task was to discriminate the checkerboard orientation. Both studies examined event-related potentials (ERPs) in response to the targets as a function of gaze direction, target location and facial expression. The authors did not observe any modulation of early components (viz. P1, N1; C1 was not analyzed in these studies) by gaze-directed attentional orienting and reported that the interaction between face emotion and gaze direction relative to target location was reflected in the P3 complex: the parieto-occipital P3 wave was reduced for valid relative to invalid targets following fearful faces [52], at least for left visual field targets [53]. No such cueing effect was observed for happy faces. However; it is possible that the design of these studies did not allow revealing any early effect of attention orienting induced by gaze. In particular, the use of dynamical facial expressions and very short cue-target asynchrony may not have favored the observation of early orienting response to the targets following the emotional gaze cues. In the present study, the P3 component could not be analyzed because our protocol was optimized for the analysis of early brain responses to targets, using a simple target detection task with reaction times of the order of 300 ms after which the subjects were allowed to blink (until the start of the next trial); accordingly, we computed ERFs only in the first 200-ms of target processing only. Therefore, we cannot relate our results to the findings of Fichtenholtz et al., and future studies will be needed to investigate the multiple stages of attention orienting effects influenced by emotion and their functional roles.

It may be noted that the early selective attention effect induced by fearful gaze was not accompanied by an enhancement of the cueing effect of gaze for fearful versus happy faces at the behavioral level. This null behavioral result may be explained by several factors. First, the increase of the gaze cueing effect (or RT difference between validly and invalidly cued targets) for fearful gaze cues has inconsistently been reported in the literature (see [25], [26] for positive results in typical Posner-like gaze cueing paradigms, and [27]–[29], [34], [52]–[55] (for null results). This suggests that the enhancement of the gaze cueing effect by associated fearful expression might be either a subtle effect or an effect difficult to uncover in reaction time measures from laboratory experiments. Second, the increase of the gaze cueing effect by fearful expression has sometimes been reported in high-anxious subjects only [25], [26]. Our sample was comprised of low-anxious participants, which may not have favoured the observation of a behavioural outcome of the impact of fearful expression on the cueing of attention by gaze. Under this perspective, magnetic brain responses turned out as more sensitive measures to uncover this impact, in the form of a very early effect of attention selective of fearful face gaze cues as compared to happy face gaze cues.

This result is reminiscent of a previous study that found a very early influence of emotion on attention processes although with a very different paradigm. Pourtois et al. (2005) [56] examined the exogenous cueing of attention by peripherally presented emotional faces. Fearful or happy faces were presented either at the location of a subsequent target bar (valid trials) or in the opposite visual hemifield. The authors found evidence for a differentiated scalp distribution of the evoked responses to validly versus invalidly cued targets between 40 and 80 ms when the peripheral cue was a fearful face; this effect involved the left posterior parietal cortex and to a lesser extent the right superior temporal cortex. Altogether, these findings and ours underline the impact of emotion on attentional processes. They demonstrate that the very early attention orienting and gain control mechanisms that allow enhancing or prioritizing the processing of validly relative to invalidly cued targets can be modulated by emotion, at least in the case of social stimuli such as faces.

Source localisation revealed the involvement of two cortical regions in the early effect of attention induced by fearful gaze: the superior parietal lobule (SPL) region, and the lateral middle occipital gyrus (MOG) region, with a left hemisphere lateralization of the activation of both regions. We will first discuss the left SPL activation. It is well known that the parietal regions are involved in attentional processes [57]. The superior parietal lobule, including in the left hemisphere, has been implicated in the control of the shifts of spatial attention [58], [59]. It is not unusual that attention paradigms activate left-lateralized activations [57] either with non social stimuli [60] or with social stimuli [4]. Furthermore, our result concords with that of Pourtois et al. 2005 [56] who found that the early (40–80 ms) topographical dissociation of the responses to targets following fearful relative to neutral faces involved the left posterior parietal cortex. In addition, a recent fMRI study of joint attention showed that the left intraparietal sulcus region is activated when an observer follows the gaze of another human [61]. The authors suggested that this region may be specifically involved in encoding dyadic relations during gaze following. A recent MEG study also reported left hemisphere activations in a paradigm of attentional orienting by neutral face gaze as well as in a paradigm of exogenous attention orienting where a central face was presented concomitantly to the peripheral attention cue [11]. The authors proposed that this left lateralization was due to the right hemisphere being busy processing the faces. Altogether, these results and ours suggest that the left lateralization of our effects may be related to the social component of our cueing paradigm. An alternative interpretation may however be proposed: Our protocol may have elicited motor attention processes [62]. Indeed, the subject’s task was a simple target detection task where he/she had to press a button as rapidly as possible as soon as the target was displayed. Thus, the subject’s attention was in fact aimed at a motor act. According to Rushworth [62]–[66], this type of attention, which he qualified as motor attention (in opposition with visual attention) may engage preferentially the left parietal regions. Thus, this may have contributed to the left lateralization of our effect. Finally, it may also be noted that our task involved a right hand response. Could this have caused the left lateralization of our effect? Although it cannot be ruled out, Rushworth’s effects were obtained even when subjects responded with the left hand. Thus, it seems more plausible that the motor component of attention and possibly the social component of our cueing paradigm explained the lateralization of our effect. Note also that the effect of the response hand could not have confounded the crucial interaction that we found between emotion and gaze because it held true for fearful as well as happy faces, and no RT differences were found between the emotions.

In addition, there were activations in the left lateral occipital region for valid versus invalid targets cued by fearful gaze. Such extrastriate activation suggests the involvement of an early gain control mechanism resulting in the amplification of the visual processing of the validly cued targets relative to the invalid (or uncued – see Figure 2) targets [67]–[69] The reason why this effect was lateralized to the left hemisphere, independently of the visual field of the target, is unclear. It is possible that local nature of the target that we used favoured left hemisphere involvement as this hemisphere is specialized for the analysis of local objects or features, especially when there is no need for precise coordinate representation [70]. Such interpretation however requires caution because it is also possible that the activations were more variable in the right hemisphere and therefore not detected.

We note that source localization did not involve significantly the striate cortex. It has long been thought that brain activities in the time range of the C1 reflected purely the initial feedforward flow of activation reaching the primary visual area, V1 [39]. However, it is now established that only the very initial part of C1 is likely to reflect mostly primary visual sources; C1 should rather be seen as reflecting an early flow of activation throughout the visual cortex encompassing V1 as well as extrastriate cortex [71]. Our results reinforce this view by showing that the early effect of attention orienting by fearful gaze in the time range of the C1 involved extrastriate visual occipital regions as well as superior parietal cortex regions.

In conclusion, the results of the present study emphasize how early the processing of encountered objects can be influenced by the social context. In doing so, it adds to the growing body of evidence of the integrated nature of gaze and emotion processing [15], [17], [22], [72], [73]. Although the early effect reported here was well upstream the simple motor act requested in our laboratory-based simple detection task, it may be associated with the adaptive value of prioritizing the processing of the objects of attention of a frightened person. This study is the first to demonstrate that the combination of gaze with fearful expression in attention orienting can impact on the earliest stages of the visual processing of ensuing targets.

## Supporting Information

Figure S1 **Results of the 2-by-2-by-2 ANOVA performed on the mean amplitude of ERFs between 55 and 70 ms, on every electrode**. The maps of the p values for the main effect of validity (a) and for the interaction between validity and emotion (b) are represented.
(TIF)

Figure S2 **a)** Topographical maps of the ERFs in response to right and left valid and invalid targets following fearful gaze cues. **b)** Topographical maps of the ERFs in response to the ‘absent targets’ following fearful gaze cues in the catch trials. **c)** Topographical maps of the ERFs in response to the uncued targets (average of right and left targets) following fearful (direct gaze) faces. For a, b, and c: The maps represent the mean amplitude of the magnetic responses between 55 and 70 ms, averaged across subjects.
(TIF)

Table S1 (DOCX)
