# Supplementary figures and images for: Watch Out! Magnetoencephalographic Evidence for Early Modulation of Attention Orienting by Fearful Gaze Cueing
Source: PLoS One. 2012 Nov 29;7(11):e50499. doi: 10.1371/journal.pone.0050499 (PMC3510181; doi:10.1371/journal.pone.0050499)

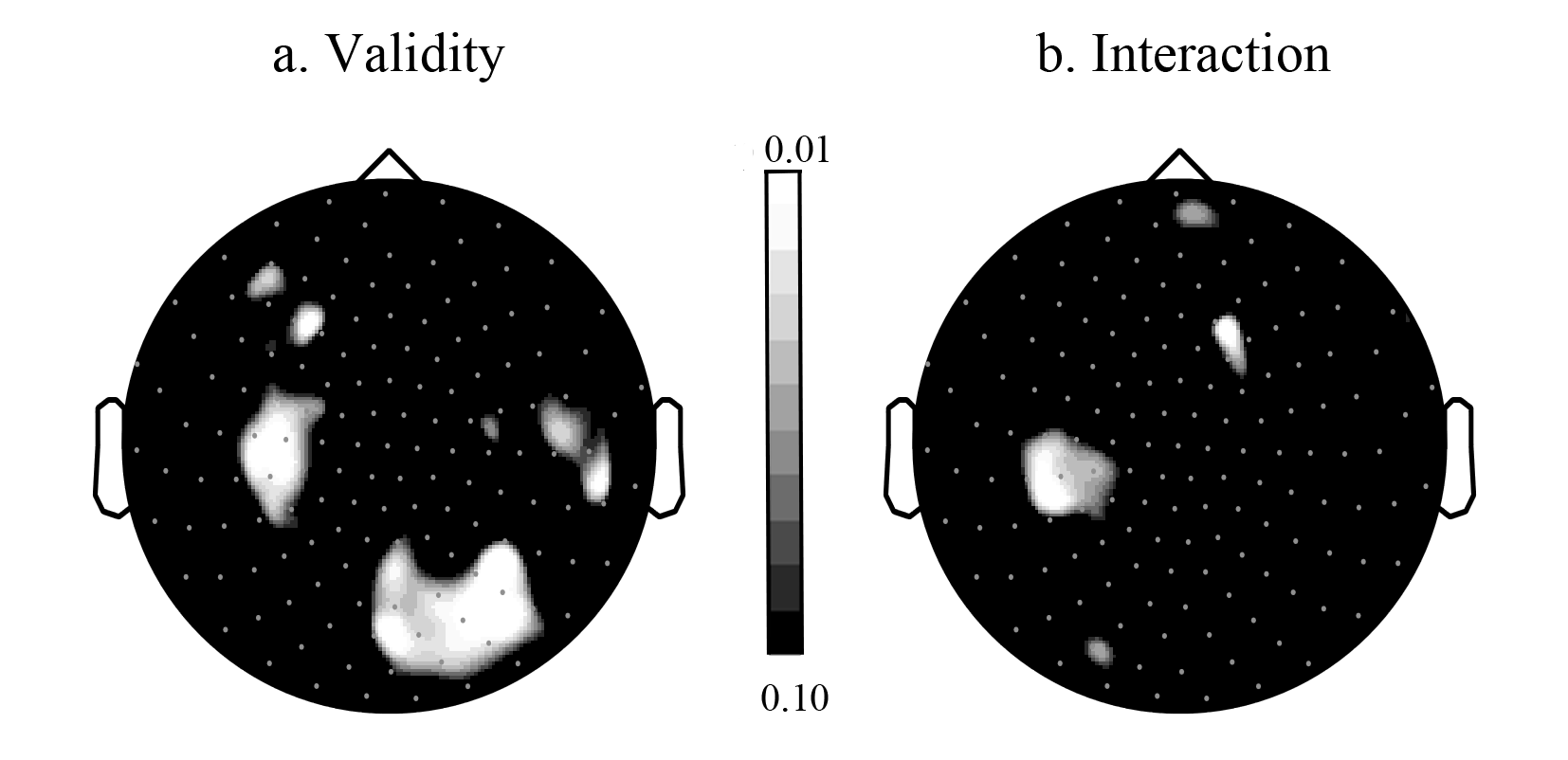

Supplement: Figure S1 — Results of the 2-by-2-by-2 ANOVA performed on the mean amplitude of ERFs between 55 and 70 ms, on every electrode. The maps of the p values for the main effect of validity (a) and for the interaction between validity and emotion (b) are represented. (TIF) [file pone.0050499.s001.tif]

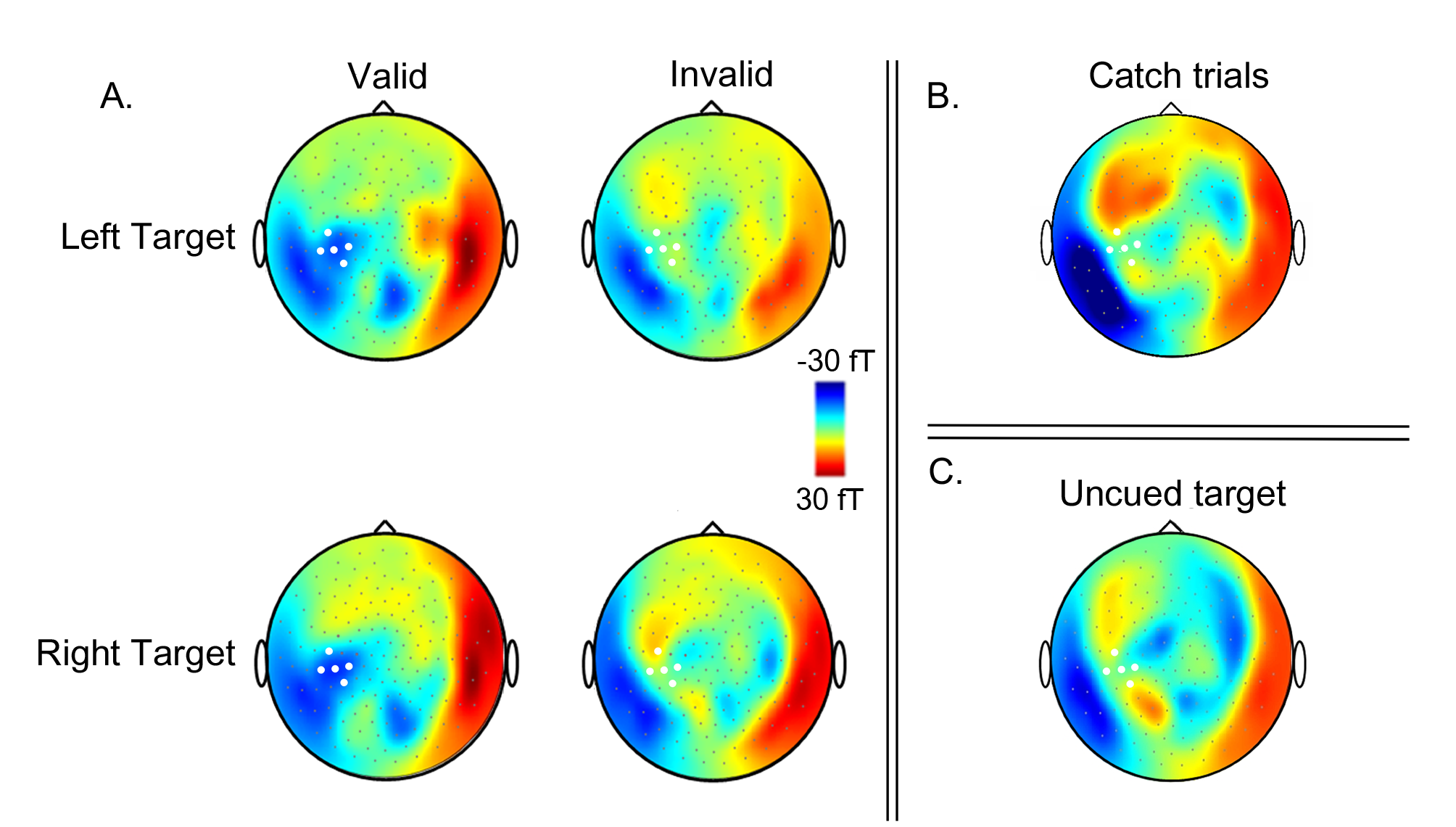

Supplement: Figure S2 — a) Topographical maps of the ERFs in response to right and left valid and invalid targets following fearful gaze cues. b) Topographical maps of the ERFs in response to the ‘absent targets’ following fearful gaze cues in the catch trials. c) Topographical maps of the ERFs in response to the uncued targets (average of right and left targets) following fearful (direct gaze) faces. For a, b, and c: The maps represent the mean amplitude of the magnetic responses between 55 and 70 ms, averaged across subjects. (TIF) [file pone.0050499.s002.tif]
