# Supplementary material for: Watch Out! Magnetoencephalographic Evidence for Early Modulation of Attention Orienting by Fearful Gaze Cueing
Source: PLoS One. 2012 Nov 29;7(11):e50499. doi: 10.1371/journal.pone.0050499 (PMC3510181; doi:10.1371/journal.pone.0050499)
Supplement: Table S1 — (DOCX) [file pone.0050499.s003.docx]

| **ANOVA** | **F value** | **p** |
| --- | --- | --- |
| Validity (Valid, Invalid, Uncued targets) | **F (2,28)=4.64** | **0.018** |
| Emotion-by-Validity interaction | **F(2,28)=3.48** | **0.044** |
|  |  |  |
| **Simple main effect of Validity:** |  |  |
| - for fearful gaze cues | **F(2,28)=7,63** | **0.002** |
| - for happy gaze cues | F(2,28)=0.082 | 0.92 |
|  |  |  |
| **Post-hoc tests: 2-by-2 contrasts of the validity conditions for each emotion** | **p value** | |
| - fearful gaze cues: |  |  |
| Valid vs. Invalid targets | **0.012** | |
| Valid vs. Uncued targets | **0.003** | |
| Invalid vs. Uncued targets | 1.000 | |
| - happy gaze cues: |  |  |
| Valid vs. Invalid targets | 1.00 | |
| Valid vs. Uncued targets | 1.00 | |
| Invalid vs Uncued targets | 1.00 | |

**Supplementary Stable.** Statistical analysis of the mean amplitude of the parietal activity in the 55-70ms time window taking into account the cue-target validity as a 3-levels factor (valid, invalid, uncued targets). In this ANOVA, Emotion (happy / fearful gaze cues) was the other within-subject factor; data were averaged across right and left targets. The significant effects are in bold characters. Post-hoc tests were Bonferroni corrected for multiple comparisons.
